# Sphingosine kinase 2-deficiency mediated changes in spinal pain processing

**DOI:** 10.3389/fnmol.2015.00029

**Published:** 2015-08-03

**Authors:** Jastrow Canlas, Phillip Holt, Alexander Carroll, Shane Rix, Paul Ryan, Lorena Davies, Dusan Matusica, Stuart M. Pitson, Claire F. Jessup, Ian L. Gibbins, Rainer V. Haberberger

**Affiliations:** ^1^Pain and Pulmonary Neurobiology, Anatomy and Histology, Centre for Neuroscience, Flinders UniversityAdelaide, SA, Australia; ^2^Centre for Cancer Biology, University of South Australia and SA PathologyAdelaide, SA, Australia

**Keywords:** sphingosine 1-phosphate, dorsal horn, RT-PCR, CFA, formalin, knock-out mouse

## Abstract

Chronic pain is one of the most burdensome health issues facing the planet (as costly as diabetes and cancer combined), and in desperate need for new diagnostic targets leading to better therapies. The bioactive lipid sphingosine 1-phosphate (S1P) and its receptors have recently been shown to modulate nociceptive signaling at the level of peripheral nociceptors and central neurons. However, the exact role of S1P generating enzymes, in particular sphingosine kinase 2 (Sphk2), in nociception remains unknown. We found that both sphingosine kinases, Sphk1 and Sphk2, were expressed in spinal cord (SC) with higher levels of Sphk2 mRNA compared to Sphk1. All three Sphk2 mRNA-isoforms were present with the Sphk2.1 mRNA showing the highest relative expression. Mice deficient in Sphk2 (Sphk2^−/−^) showed in contrast to mice deficient in Sphk1 (Sphk1^−/−^) substantially lower spinal S1P levels compared to wild-type C57BL/6 mice. In the formalin model of acute peripheral inflammatory pain, Sphk2^−/−^ mice showed facilitation of nociceptive transmission during the late response, whereas responses to early acute pain, and the number of c-Fos immunoreactive dorsal horn neurons were not different between Sphk2^−/−^ and wild-type mice. Chronic peripheral inflammation (CPI) caused a bilateral increase in mechanical sensitivity in Sphk2^−/−^ mice. Additionally, CPI increased the relative mRNA expression of P_2_X_4_ receptor, brain-derived neurotrophic factor and inducible nitric oxide synthase in the ipsilateral SC of wild-type but not Sphk2^−/−^ mice. Similarly, Sphk2^−/−^ mice showed in contrast to wild-type no CPI-dependent increase in areas of the dorsal horn immunoreactive for the microglia marker Iba-1 and the astrocyte marker Glial fibrillary acidic protein (GFAP). Our results suggest that the tightly regulated cell signaling enzyme Sphk2 may be a key component for facilitation of nociceptive circuits in the CNS leading to central sensitization and pain memory formation.

## Introduction

Pain, including chronic pain is a major health problem that greatly reduces the quality of life for the patient and represents not only a significant social but also economic burden. Management is poor due to lack of reliable diagnostic tools to identify specific causes of chronic pain combined with few and frequently insufficient treatments. Consequently, there is an urgent unmet medical need for both new diagnostic tools and better therapies for chronic inflammatory pain. However this quest is confounded since there are not *one* but multiple pathologies and affected signaling pathways that eventually lead to the chronic inflammatory, neuropathic or pathological pain.

Pain is accompanied by activation of a chain of nociceptive neurons in dorsal root ganglia (DRG), spinal cord (SC) and the brain that is modulated by the release of inflammatory mediators from SC glia as part of tri-partite communication between microglia, astrocytes and neurons (Liang et al., 2013). The precise molecular mechanisms regulating this communication between cells remain an important, but unanswered question. One molecule that has recently been shown to be involved in neuronal pain signaling as well as in the function of glia is the bioactive sphingolipid sphingosine 1-phosphate (S1P; Muscoli et al., 2010; Mair et al., 2011; Camprubí-Robles et al., 2013; Finley et al., 2013; Salvemini et al., 2013; Janes et al., 2014). S1P is generated by the phosphorylation of sphingosine by two sphingosine kinase isoforms (Sphk1; Sphk2). It can function both as an extracellular ligand for a family of five G-protein coupled S1P receptors (S1PR1-5), and as an intracellular messenger through interactions with and regulation of different cytosolic and nuclear proteins (for review, see Pitman and Pitson, 2010; Pitson, 2011). The Sphk1 and Sphk2 proteins differ in size, intracellular localization and substrate specificity (Bryan et al., 2008; Liu et al., 2012; Orr Gandy and Obeid, 2013). Sphk1 is a cytosolic protein that upon activation is phosphorylated by the Erk1/2 kinases and relocates to the plasma membrane, whereas Sphk2 is localized to the endoplasmatic reticulum, mitochondria and has a nuclear localization sequence which allows relocation into the nucleus (Siow and Wattenberg, 2011; Strub et al., 2011; Orr Gandy and Obeid, 2013). Interestingly, Sphk2 but not Sphk1 phosphorylates the drug FTY720 (fingolimod, Gilenya^TM^) into its active form which has been shown to modulate inflammatory and neuropathic pain signaling (Coste et al., 2008b; Janes et al., 2014; Zhang et al., 2015).

Intracerebroventricular injection of S1P produced antinociceptive effects (Sim-Selley et al., 2009) and acute and Chronic peripheral inflammation (CPI) reduced the S1P content in the SC (Coste et al., 2008a). Blockade of spinal S1P1 receptor subtype blocked neuropathic pain (Janes et al., 2014). In the present study we investigated the impact of Sphk2-deficiency in acute and chronic inflammatory pain. Based on our novel findings of changed nocifensive behavior in Sphk2-deficient mice in response to acute and chronic inflammation we focused on S1P levels, mRNA and protein expression and level of the SC dorsal horn as it is an essential area in pain processing and in the development of central sensitization. Our results indicate that the tightly regulated cell signaling enzyme, Sphk2, may be a key component for facilitation of nociceptive circuits in the CNS eventually leading to central sensitization and pain memory formation.

## Materials and Methods

### Animals

Male 6–10 weeks old Sphk1^−/−^ (Allende et al., 2004), Sphk2^−/−^ (Mizugishi et al., 2005) or C57BL/6 (wild-type, wt) mice (Flinders Medical Centre Animal house) were used. Mice were housed on a 12 h light/dark cycle with free access to mouse chow and water. All procedures were approved by the Animal Welfare Committee of the Flinders University.

### Formalin-Induced Nocifensive Behavior

For the formalin test (Sun et al., 2013), mice were placed in plexiglas chambers and left for 15–30 min for acclimatization. The formalin solutions (0.5 and 2%) were freshly prepared from 37% formaldehyde solution which equals 100% formalin (Merck) at the day of injection (Sun et al., 2013). During a short isoflurane-induced anesthesia 15 μl formalin (2%) solution was injected into the left plantar hindpaw. 0.5% formalin was injected subcutaneously into the dorsal hindpaw (Shields et al., 2010). Injection of sterile saline 0.9% served as control. Subsequently the mouse behavior was recorded for 45 min. The recordings were analyzed for the time spent licking, grooming, exploring or the time without any visible movement. The total amount of time spent for individual behaviors was separated into 5 min intervals. The investigator was blinded in respect to the strains used.

### Complete Freund’s Adjuvant (CFA)-Induced Mechanical Hyperalgesia

Mechanical withdrawal thresholds in response to CPI were evaluated according to a previously described method (da Costa et al., 2010). The animals received 20 μl of complete Freund’s adjuvant (CFA) injected intra plantar in the left hindpaw. The paw withdrawal behavior was evaluated 1 h before injection (baseline measure) and at different time-points (4, 24, 48 and 168 h) following CFA injection. Mice (at least one C57BL/6-wt and one Sphk2-deficient mouse per experiment) were placed individually in plexiglas container (17.5 × 17.5 × 12 cm) with four compartments, on an elevated wire mesh platform. The investigator was blinded with respect to strains used. The animals were acclimatized for 30 min prior to behavioral testing. The frequency of withdrawal response (%) was measured following 10 applications of von Frey Hairs onto the plantar surface of the left and right hindpaw (with a duration of 3–5 s for each application). Von Frey hairs from 0.0275 to 1.202 g were used. At days 3 and 7 the thickness of the mid-third of left (ipsilateral) and right (contralateral) hindpaw was measured using a caliper. The paw withdrawal threshold of individual mice was determined at the von Frey hair intensity at which there was a minimum 50% positive incidences of pain-related behavior (out of 10 applications of the von Frey hair). In the event that the von Frey hair did not reach 50%, the next stronger filament was used; alternatively, in the event of a positive withdrawal response, the next weaker stimulus in the series was used. The paw withdrawal threshold at which mice responded to 50% of mechanical stimuli prior to and following CFA injection were used to compare strains and their response to peripheral inflammation.

### Determination of Sphk1 Activity and S1P Content in Spinal Cord Tissue

Lysis buffer composed of 50 mM Tris/HCl (pH 7.4), 150 mM NaCl, 2 mM activated Na_2_VO_3_, 10 mM NaF, 10 mM β-glycerophosphate, 1 mM EDTA, 1 mM DTT, 10% glycerol, 0.05% Triton X-100 and Complete^TM^ protease inhibitor cocktail (Roche) was added to SC tissue in an approximate 1:1 ratio. The tissue was then homogenized with a microtube pestle (Axygen), followed by 4 × 30 s cycles of sonication on ice in a Bioruptor bath sonicator (Diagenode, NY, USA). The lysate was then assayed for Sphk1 activity using D-*erythro*-sphingosine and [γ^32^P]ATP as substrates (Pitman et al., 2012), and S1P content by high performance liquid chromoatography with post-column fluroescent derivatization, as previously described (Leclercq et al., 2011). Protein concentrations in the lysates were determined using the Bio-Rad protein assay reagent and bovine serum albumin as standard.

### Isolation of Total RNA and Reverse Transcription

Lumbosacral SC was dissected, ipsi- and contralateral sides separated and stored in Trizol (Sigma) at −80°C. Samples were homogenized using a tissue lyser (Qiagen). Total RNA was isolated using a column based method (Zymo-Spin ICC Columns, Zymogen, Irvine, CA, USA). DNA contamination was removed by on-column DNA digestion. The concentration of total RNA was determined using standard photospectrometry (Nanodrop 2000, Thermo Scientific, Australia), quality of RNA was determined using a lab-on-chip system (Bioanalyser, Agilent). Only samples with RNA integrity numbers (RIN) above seven were used for subsequent analysis. One microgram of total RNA was reverse transcribed into cDNA according to the manufacturer protocol (SuperscriptII, BioRad, Australia).

### qPCR

Quantitative polymerase chain reaction (qPCR) analysis of the relative mRNA expression levels in the DRG samples was performed using the StepOnePlus cycler (Life Technologies). TaqMan primers (Life Technologies) were used for the detection of pro-inflammatory markers and molecules characteristic for microglia. Beta-2 microglobulin (B2M) was used as a reference gene (Vandesompele et al., 2002). The efficiencies of all primer-pairs were determined by 1/5 to 1/625 dilutions in a qPCR and a satisfying efficiency was determined with Q-Gene (Simon, 2003). The primers and efficiencies are listed (Table 1). The final volume for qPCR was 20 μl of which 8 μl were H_2_O, 10 μl mastermix (Life Technologies), 1 μl assay-mix (Life Technologies) and 1 μl cDNA. Each qPCR was done in duplicate. The Ct values were determined for each product and normalized as pairwise comparisons against the Ct value of the reference gene. Subsequently the mean normalized expression (MNE) was calculated and differences in the expression determined (Simon, 2003).

**Table 1:** **TaqMan primer assays**.

Primer assay	Assay code	Amplicon length (bp)	Efficiency (R2)
B2M	Mm00437762_ml	77	0.9997
Sphkl	Mm00448841_gl	65	0.9830
Sphk2	Mm00445021_ml	113	0.9977
Sphk2.1	Mm00445020_ml	133	0.9972
Sphk2.2	Mm00772700_ml	85	0.9959
Sphk2.3	Mm01204085_ml	62	0.9949
P2X4	Mm00501787_ml	66	0.9944
NOS 2 (iNOS)	Mm00440502_ml	66	0.9891
Bdnf	Mm01334042_ml	108	0.9891
Il-1β	Mm00434228_ml	90	0.9997
Il-6	Mm00446190_ml	78	0.9975

*The table shows the assay used for qRT-PCR experiments with abbreviations for the mRNAs and mRNA isoforms, assay codes, length of the PCR products in base pairs (bp) and the tested primer efficiencies*.

### Western Blot

Whole SCs and DRG were lyzed using chilled radioimmunoprecipitation (RIPA) lysis buffer containing 10 mM Tris-HCl, pH 8.0, 150 mM NaCl, 2 mM EDTA, 1% NP-40, 1% Triton X-100, 10% glycerol, 1 mM phenylmethanesulfonyl fluoride, 1 mM sodium orthovanadate, 1 μM batimastat (BB-94), and 1% protease inhibitor cocktail (Roche). For Western blots, lysates were solubilized in an equal volume of 2 × SDS sample buffer containing 4% SDS, 2% glycine, 0.015% bromophenol blue, 20% glycerol, and 10% β-mercaptoethanol in 100 mM Tris-HCl buffer, pH 6.8. Cell lysates were electrophoresed through 4–12% Bis-Tris buffered SDS gels (Life Sciences). Proteins were transferred onto poly(vinylidene fluoride) (PVDF) membrane at 100 V for 1 h. The membranes were blocked in 4% skim milk powder, 0.1% Tween-20, and 0.02% NaN_3_ in theta burst stimulation (TBS), pH 8.0, for 1 h at room temperature and incubated overnight with primary antisera. The following antibodies were used: rabbit anti-SphK2 (1:500; S Pitson, Adelaide, SA, Australia) and mouse anti beta-III tubulin (1:1000; Promega). Membranes were washed three times in TBS-Tween 20 (TBST), pH 8.0, for 10 min and incubated for 1 h with donkey anti-rabbit horseradish peroxidase (HRP) or donkey anti-mouse HRP secondary (1:50,000; Invitrogen) in TBS at room temperature and subsequently washed three times in TBS for 10 min and developed with Supersignal West Pico Sensitivity Substrate (Pierce).

### Multiple Labeling Immunohistochemistry

For immunohistochemical analysis, animals were euthanized with an overdose of isoflurane and 20 mm of the lumbosacral SC covering entry area of the dorsal roots L3-L5 was dissected and fixed by immersion in Zamboni’s fixative (2% formaldehyde; 0.5% picric acid; 0.2 M sodium phosphate buffer, pH 7.0) at 4°C for 24 h. Subsequently, the SC was dehydrated through a graded series of ethanol and DMSO, embedded in polyethylene glycol (PEG, 1450 MW: Sigma-Aldrich, St. Louis, MO, USA; Murphy et al., 1998) and the complete lumbosacral region adjacent to dorsal roots that connect to the sciatic nerve was sectioned at 12 μm with 10 sections (120 μm) between sections. Free floating sections were short term stored at 4°C in PBS-sodium azide or immediately used for multiple labeling immunohistochemistry.

The sections were blocked with 10% normal donkey serum in PBS for 30 min and subsequently incubated for 48 h in a mixture of primary antisera (Table 2), diluted in hypertonic PBS containing 10% normal donkey serum under humid conditions. After washing in PBS, sections were incubated for 2 h in a mixture of secondary antisera (Table 2). After a final PBS wash, the sections were coverslipped in carbonate-buffered glycerol (pH 8.6). Microglia was labeled using an antiserum directed against calcium-binding adapter molecule, Iba-1, astrocytes were detected using antiserum directed against GFAP, neurons were labeled with NeuN (Table 2). 4′,6-diamidino-2-phenylindole (DAPI) was used to stain nuclei.

**Table 2:** **Primary and secondary antisera**.

Primary antisera	Source	Host animal	Dilution	Secondary antisera	Dilution
GFAP	Novacastra	Mouse	1:200	Donkey anti mouse IgG FITC conjugated	1:50
Iba-1	AbCam	Goat	1:200	Donkey anti sheep IgG Cy5 conjugated	1:50
NeuN	Chemicon	Mouse	1:200	Donkey anti mouse IgG FITC conjugated	1:50
cFOS	Santa Cniz	Rabbit	1:600	Donkey anti rabbit IgG Cy3 conjugated	1:100
DAPI	Sigma				1:1000

*The table shows the used combinations of primary and secondary antisera with dilutions and suppliers*.

To investigate the formalin-dependent activation of cells in the SC dorsal horn we used an antiserum directed against the proto-oncogene Finkel-Biskis-Jinkins (FBJ) murine osteosarcoma viral oncogene homolog, c-Fos (Table 2).

### Image Analysis

Images of sections with different immunoreactivites were taken using a BX-50 fluorescence microscope (Olympus, Australia). Images were imported into ImageJ (NIH, Bethesda, MD, USA) for subsequent analysis.

The dorsal horn was defined by the characteristic arrangement and density of DAPI-stained nuclei the area measured and used for subsequent analyses. Using triple-labeled images we determined the total number nuclei (Dapi), of NeuN-positive cells and the areas occupied by immunoreactivities for Iba-1 or GFAP. The areas and cell numbers were normalized to 100,000 μm^2^ to adjust for differences in measured areas of dorsal horn. Numbers of nuclei positive for c-Fos in the dorsal horn were counted manually and each positive c-FOS staining was checked for immunoreactivity (IR) to the nuclear marker Dapi. Normally 3–4 sections per animal were used for the analysis of c-Fos IR in response to acute peripheral inflammation in response to formalin injection.

For analysis of the CPI13–18 sections with 100 μm in between sections covering the entire lumbosacral entry zone of dorsal roots L3–5 were analyzed per animal to determine differences in the immunoreactive areas positive for GFAP and Iba-1. To correct for differences in staining intensity between animals and sections, for each section the contralateral side was set as 100% and IR on the ipsilateral side determined in comparison to the contralateral side.

### Statistical Analysis

For the analysis of relative mRNA expression, responses to formalin and mechanical hypersensitivity, we used one- or two-way Analysis of Variance (two-way ANOVA, GraphPad Prism 5, San Diego, CA, USA). If the resulting *F*-value was significant (*P* < 0.05) for the responses to formalin and mechanical hypersensitivity, then *post hoc t* tests with Bonferroni correction were performed to obtain individual comparisons at each time point, and significant differences are denoted in figures. For analysis of immunoreactivities we used paired and unpaired Students *t*-test (GraphPad Prism 5). All data are presented as mean ± SEM.

## Results

### Sphk Expression in Spinal Cord

**Figure 1:**
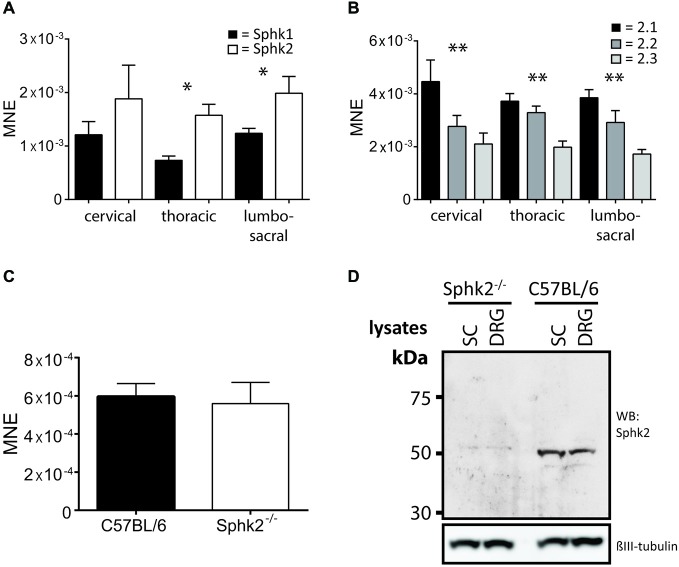
**Expression of sphingosine kinases in mouse spinal cord (SC)**. Relative mRNA-expression levels as mean normalized expression (MNE) for **(A)** the two sphingosine kinase isoform, Sphk1 and Sphk2, at different levels of the mouse SC. Sphk2 is expressed significantly higher compared to the Sphk1 isoform in thoracic and lumbosacral SC levels (*n* = 5, one-way ANOVA, *p < 0.05); **(B)** the three Sphk2 mRNA-isoforms at different levels of the mouse SC. The isoforms were significantly different expressed at all SC levels with Sphk2.1 isoform showing the highest relative mRNA-expression (*n* = 5, one-way ANOVA, Bonferroni’s *post hoc* test, **p < 0.01); **(C)** the Sphk1isoform in SC of C57BL/6 and Sphk2^−/−^ mice. The relative expression levels were not different between strains (*n* = 5, *t*-test). Values are expressed as mean ± SEM. **(D)** Western Blot confirmed the absence of the Sphk2 protein in Sphk2-deficient mice. Sphk2 protein was present in SC and DRG and could be detected at a molecular weight of about 50 kDa. βIII tubulin used as reference.

To determine the presence and relative expression values for Sphks at the transcriptional level we determined the relative mRNA expression at different SC levels in C57BL/6-wt mice. In comparison, the relative mRNA expression levels were significantly higher for Sphk2 compared to Sphk1 (*n* = 5, one-way ANOVA, *p* < 0.05, Figure 1A) with significantly higher expression of Sphk2 compared to Sphk1 in thoracic and lumbosacral SC (*n* = 5, *t*-test, *p* < 0.05). Three Sphk2 mRNA isoforms are present in mice that differ in their 5′ untranslated regions, but are translated into identical proteins. Interestingly, all three Sphk2 mRNAs were significantly differently expressed (*n* = 5, one-way ANOVA, *p* < 0.01) with the Sphk2.1 mRNA transcript showing the highest relative expression level at cervical, thoracic and lumbosacral levels, followed by Sphk2.2 and Sphk2.3 (Figure 1B). Analysis of total RNA from lumbosacral SC showed no difference in relative mRNA expression levels for Sphk1 between Sphk2^−/−^ and C57BL/6-wt mice (*n* = 5, *t*-test, Figure 1C). Sphk2 mRNA could not be detected in total RNA extracted from Sphk2^−/−^ mouse tissue (data not shown).

The presence of the Sphk2 protein in wild-type C57BL/6 SC and DRG and its absence in Sphk2^−/−^ mouse tissue was confirmed using Western blot (Figure 1D).

### The S1P Content in the Spinal Cord is Reduced but the Sphk1-Activity Unaltered in Sphk2-Deficient Mice

**Figure 2:**
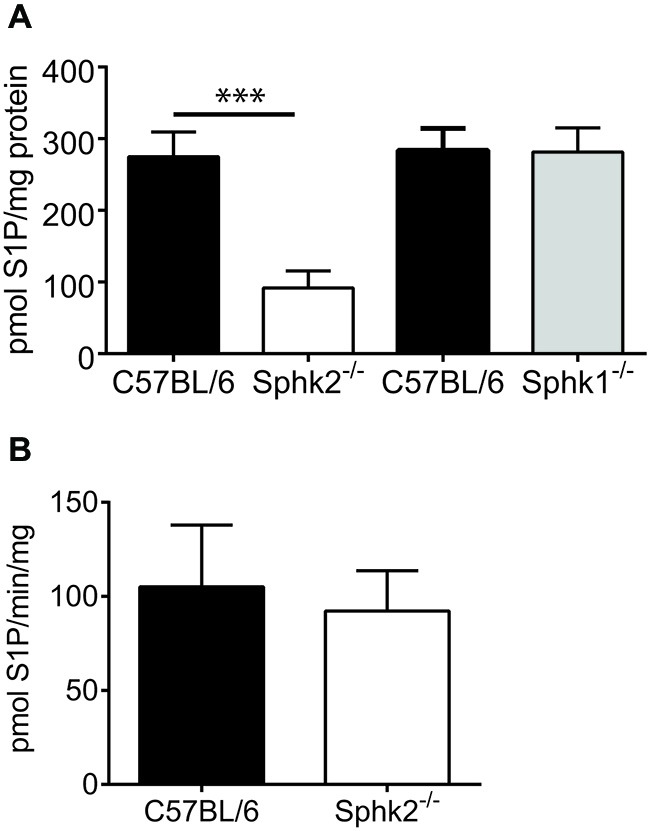
**Sphingosine kinase activity and sphingosine 1-phosphate (S1P) levels in mouse SC. (A)** Assessment of the amount of spinal S1P in C57BL/6 and Sphk-deficient mice. Whereas the amount of S1P was substantially reduced in SCs from Sphk2^−/−^ mice in comparison to C57BL/6 it was virtually unchanged in Sphk1^−/−^ mice (*n* = 5, one-way ANOVA, Bonferroni’s *post hoc* test, ***p < 0.001). **(B)** The Sphk1 activity was not different between SCs of C57BL/6 and Sphk2^−/−^ mice (*n* = 5, paired *t*-test).

To investigate Sphk function, we determined the amount of Sphk2-generated S1P in the SC. To rule out compensatory mechanisms due to the presence of Sphk1 we analyzed in addition to S1P content also Sphk1-activity in the SC of Sphk2^−/−^ and C57BL/6-wt mice (*n* = 5). The SC of Sphk2^−/−^ mice contained a significantly lower amount of S1P compared to C57BL/6-wt mice (paired *t*-test, *p* < 0.002, Figure 2B) whereas the Sphk1-activity was not different between C57BL/6-wt and Sphk2^−/−^ mice (paired *t*-test, Figure 2B). This indicates that Sphk2 is the primary source of S1P in the SC and that there is no compensatory regulation of Sphk1 in the absence of Sphk2. The data are supported by the fact that the spinal S1P content was virtually unchanged in Sphk1^−/−^ mice (Figure 2A). Unchanged Sphk2 activity in Sphk1^−/−^ has been shown recently (Beroukas et al., 2015).

### Sphk2-Deficiency Modulates the Second but not First Phase of the Response to Acute Inflammation

**Figure 3:**
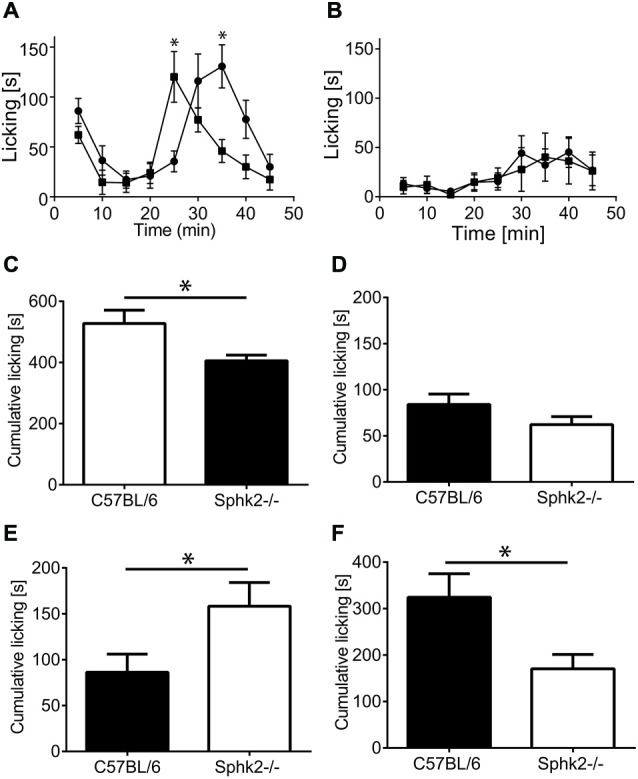
**Licking response to acute peripheral inflammation induced by formalin. (A)** Time course of the response of C57BL/6 mice (black squares) and Sphk2^−/−^ mice (black circles) to injection of 2% formalin. Time points represent mean ± SEM response of eight animals during 5 min intervals. Asterisks indicate significantly greater time licking (*p < 0.05, two-way ANOVA, Bonferroni’s test). **(B)** Time course of the response of C57BL/6 mice (black squares) and Sphk2^−/−^ mice (black circles) to injection of 0.5% formalin. The response to injection of 0.5% formalin was not different between strains. **(C–F)** Cumulative paw licking time in intervals. Values represent mean ± SEM. **(C)** Overall time spent licking in response to 2% formalin (unpaired *t*-test, *p < 0.05). **(D)** Overall response to 0.5% formalin. **(E)** Time spent licking between 10–25 min (unpaired *t*-test, *p < 0.05). **(F)** Time spent licking between 25–45 min (unpaired *t*-test, *p < 0.05).

We used the formalin model to investigate differences in the responses to acute inflammation by determining the 1^st^ and 2^nd^ phase of the licking response between animals with deficiency in Sphk2 and C57BL/6-wt. There was a significant difference in the 2nd phase in response to injection of 2% formalin (two-way ANOVA, Bonferroni’s *post hoc* test, *n* = 8, Figure 3A) whereas the response to injection of 0.5% formalin was not different between strains (Figure 3B). Sphk2^−/−^ mice responded significantly earlier with an increase in time spent licking (Figure 3A) which was confirmed by comparison of cumulative licking time from 10–25 min post formalin injection (Figure 3F). But C57BL/6-wt mice spent significant more time licking from 25–45 min (Figure 3F) and overall more time (Figure 3C, unpaired *t*-test, *p* < 0.05). The time course of the 1^st^ phase of licking response was not different between strains and the overall time spent licking within the first 10 min was not different (Figures 3A,D).

### Acute Peripheral Inflammation Increases Immunoreactivity for the Astrocyte Marker GFAP in the Ipsilateral Dorsal Horn of Sphk2^−/−^ and Wild-type C57BL/6 Mice

**Figure 4:**
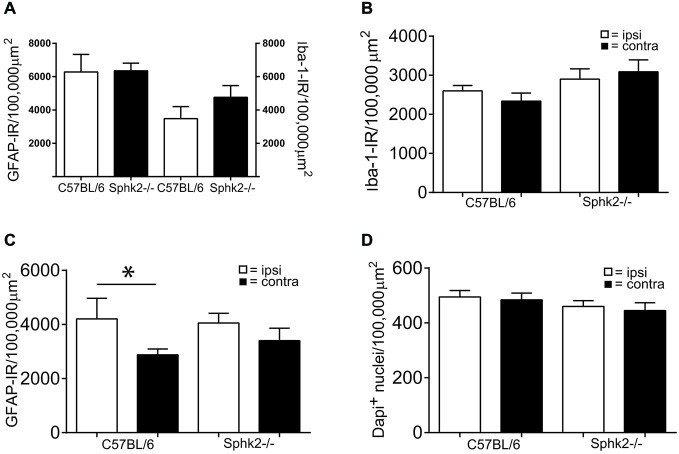
**Microglia and astrocytes in the dorsal horn in C57BL/6 and Sphk2^−/−^ mice. (A)** Areas with immunoreactivity (IR) for the astrocyte marker GFAP and the microglia marker Iba-1 in the dorsal horn of untreated C57BL/6 and Sphk2^−/−^ mouse SC normalized to 100,000 μm^2^ (*n* = 5, unpaired *t*-test, n.s.). **(B)** Area with IR for Iba-1 in the dorsal horn of C57BL/6 and Sphk2^−/−^ mouse SC 60 min post injection of two percent formalin, normalized to 100,000 μm^2^ (*n* = 10, unpaired *t*-test, n.s.). **(C)** Area with IR for GFAP in C57BL/6 and Sphk2^−/−^ mouse SC dorsal horn 60 min post injection of two percent formalin, normalized to 100,000 μm^2^ (*n* = 10, unpaired *t*-test, *p < 0.05). **(D)** Number of nuclei immunoreactive for NeuN in C57BL/6 and Sphk2^−/−^ mouse SC dorsal horn 60 min post injection of two percent formalin, normalized to 100,000 μm^2^ (*n* = 10, unpaired *t*-test).

We considered that the behavioral differences might have been related to differences in the cellular composition of the dorsal horn. To investigate differences in the cellular composition related to Sphk2-deficiency, we used multiple labeling immunohistochemistry to compare overall cell number, number of neurons and presence of astrocytes and microglia. We compared the number of DAPI-positive nuclei, the number of NeuN-positive cells and areas occupied by IR for GFAP or Iba-1 and in the SC dorsal horn between strains in response to saline, formalin and in mice without treatment. The total number of DAPI-positive nuclei, NeuN-immunoreactive neurons and areas with IR for Iba-1 were not different between strains in absence of treatment (Figure 4A) and did not change in the response to treatment (Figures 4B,D). This indicates no loss of neurons or microglia in Sphk2-deficient adult SC. Surprisingly, in both strains a larger area of the dorsal horn was occupied by clear GFAP-IR on the ipsilateral compared to the contralateral side 60 min after injection of 2% formalin (*n* = 10, *t*-test, *p* < 0.05, Figure 4C).

### Acute Peripheral Inflammation Increases the Number of c-Fos Positive Neurons

**Figure 5:**
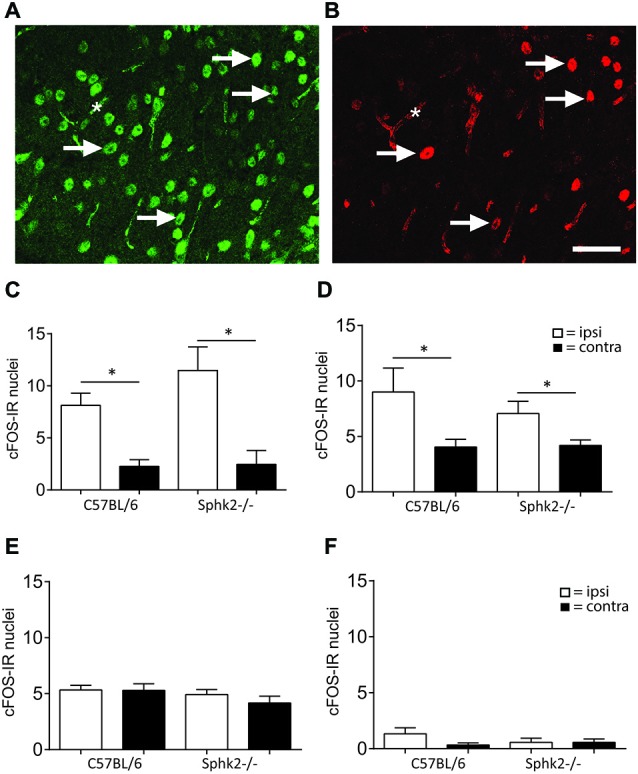
**IR for c-Fos in the dorsal horn of C57BL/6 and Sphk2^−/−^ mice in response to acute peripheral inflammation. (A,B)** Double- labeling immunohistochemistry for NeuN and c-Fos. Nuclei with immuno-reactivity for NeuN **(A)** and c-Fos **(B)** in the ipsilateral SC dorsal horn after injection of formalin into the hindpaw. c-Fos positive nuclei also showed IR for NeuN. The arrows indicate some double-labeled nuclei. The asterisk indicates background labeling of capillaries. Bar = 50 μm. **(C)** Number of nuclei with IR for c-Fos in the SC dorsal horn of C57BL/6 and Sphk2^−/−^ mice 60 min after injection of two percent formalin. **(D)** Number of nuclei with IR for c-Fos in the SC dorsal horn of C57BL/6 and Sphk2^−/−^ mice 60 min after injection of 0.5% formalin. **(E)** Number of nuclei with IR for c-Fos in the SC dorsal horn of C57BL/6 and Sphk2^−/−^ mice 60 min after injection of saline. **(F)** Number of nuclei with IR for c-Fos in the SC dorsal horn of C57BL/6 and Sphk2^−/−^ mice in untreated SC. Values represent mean ± SEM, *n* = 5, one-way ANOVA, Bonferroni’s *post hoc* test, *p < 0.05.

To investigate if the behavioral differences are related to difference in the activation of dorsal horn neurons, we determined the number of c-FOS positive neurons in the SC dorsal horn. Injection of 2 and 0.5% formalin into the left hindpaw increased the number of nuclei with IR for c-Fos in the lumbar SC of both strains. The IR was present in laminae I and II and in a small number of nuclei in deeper lamiae and was restricted to NeuN–immunoreactive neurons (Figures 5A,B). The number of c-Fos immunoreactive neurons increased significantly on the ipsilateral side in response to acute peripheral inflammation induced by 2 and 0.5% formalin (*n* = 5, one-way ANOVA, Figure 5). However, untreated or saline injected mice, stained only occasionally for c-Fos positive nuclei were present in the dorsal horn (Figures 5E,F). The increase and number of c-Fos positive neurons was not significantly different between strains.

### Sphk2-Deficiency Increases Mechanical Sensitivity in Response to Chronic Inflammation in the ipsi- and Contralateral Hindpaw

**Figure 6:**
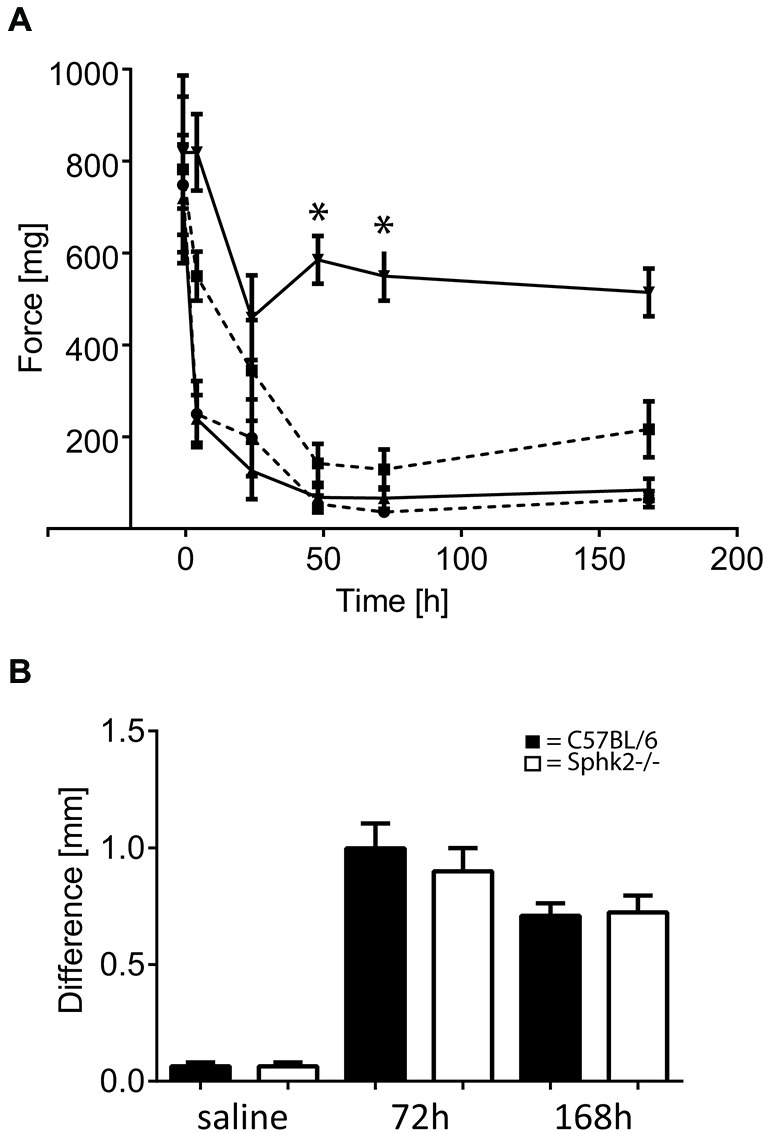
**Paw withdrawal thresholds in response to chronic peripheral inflammation (CPI) induced by CFA in C57BL/6 and Sphk2^−/−^ mice. (A)** Time course of paw withdrawal thresholds in response to intraplantar injection of CFA in C57BL/6 (*n* = 8, ipsilateral, upwards pointing triangles solid line; contralateral downward pointing triangles solid line) and Sphk2^−/−^ (*n* = 8, ipsilateral, circles dashed line; contralateral squares dashed line) hindpaws (two-way ANOVA, Bonferroni’s *post hoc* test, *p < 0.05). **(B)** Difference in mid-hindpaw thickness of the between ipsi- and contralateral side in response to CFA injection. Values represent mean ± SEM.

The observed nocifensive behavior differences in response to acute inflammation suggested that absence of Sphk2 changes the nociceptive processing at the level of the SC. To examine the effects of Sphk2-deficiency on the response to CPI we analyzed the behavior in response to mechanical stimulation. There was no significant difference in paw withdrawal threshold between the ipsilateral and contralateral hind paws prior to CFA injection (Figure 6A). The injection of CFA in hindpaws of C57BL/6 mice caused a persistent significant reduction of the mean paw withdrawal threshold ipsilateral to the injection site starting after 4 h and still present on day 7 (*n* = 8, two-way ANOVA, Bonferroni’s *post hoc* test, *p* < 0.05, Figure 6A). A significant interaction was present between affected site and measurement (two-way ANOVA, *p* < 0.0001).

Mice with deficiency in Sphk2 showed a similar reduction in mechanical threshold of the ipsilateral hind paw in response to CFA (*n* = 9, two-way ANOVA, Bonferroni’s *post hoc* test, *p* < 0.0001, Figure 6A) compared to baseline before injection. Surprisingly, the contralateral side showed also a strong reduction in mechanical threshold that was not different from the ipsilateral side but was significantly different from the contralateral side in C57BL/6-wt animals (*n* = 8, two-way ANOVA, Bonferroni’s *post hoc* test *p* < 0.0001, Figure 6A).

Analysis of the paw oedema showed no differences between C57BL/6-wt- and Sphk2^−/−^ mice (Figure 6B). The ipsilateral paws showed similar increase in thickness of the mid-hindpaw in response to saline and CFA injection after 3 and 7 days (*n* = 7–10 per condition and strain) and daily observation showed no differences in the behavior or the extent of inflammation.

### S1P Levels in Spinal Cord in Response to Chronic Inflammation

**Figure 7:**
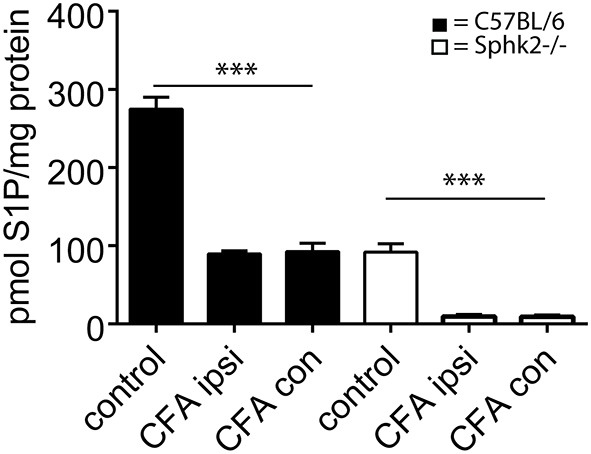
**S1P content of ipsi- and contralateral SC in response to CPI induced by CFA in C57BL/6 and Sphk2^−/−^ mice**. Analysis of the amount of S1P in C57BL/6 and Sphk-deficient mice at 7 days post-CFA injection into the hindpaw. The amount of S1P was substantially reduced in ispi- and contralateral SCs from C57BL/6 and Sphk2^−/−^ mice in comparison to untreated controls (control, *n* = 5, ispi- and contralateral SC *n* = 4, one-way ANOVA, Bonferroni’s *post hoc* test, ***p < 0.001).

As these behavioral differences and the presence of bilateral hypersensitivity might be reflected by differences at the level of S1P synthesis, we analyzed the S1P content in ipsi- and contralateral SC in mice 7 days after a CFA injection into the left hindpaw. No differences were observed between ipsi- and contralateral S1P content (*n* = 4) in both strains. However similar to non-inflamed mice, Sphk2^−/−^ mice had significantly lower SC S1P levels compared to C57BL/6 mice (Figure 7, one-way ANOVA, Bonferroni’s *post hoc* test, *p* < 0.0001).

### Sphk2-Deficiency Reduces Spinal mRNA Expression of P2X4, Bdnf and Nos2 in Response to Chronic Inflammation

**Figure 8:**
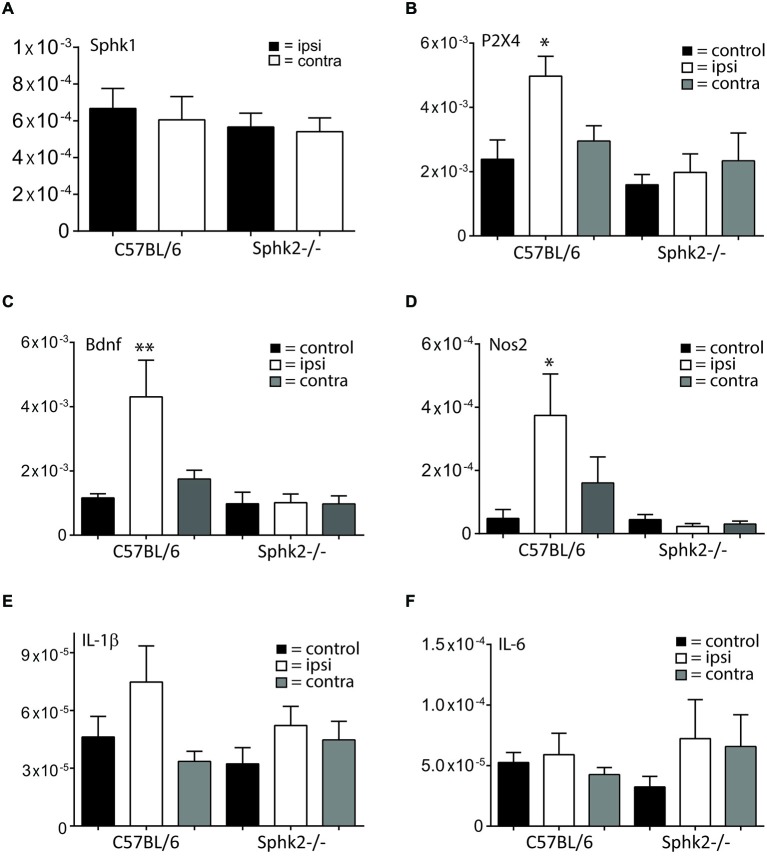
**Relative mRNA expression in ipsi- and contralateral SC in response to CPI**. Relative mRNA expression levels in the SC of C57BL/6 mice and Sphk2-deficient mice (Sphk2^−/−^). The data are presented as MNE. **(A)** Relative mRNA-expression levels for sphingosine kinase 1 (Sphk1) in ipsi- and contralateral lumbosacral SC of C57BL/6 and Sphk2^−/−^ mice after 7 days CFA. **(B)** Relative mRNA-expression levels for the P_2_X_4_ receptor (P2X4) in lumbosacral SC of untreated control mice (control) and in ipsi- (ipsi) and contralateral (contra) lumbosacral SC of C57BL/6 and Sphk2^−/−^ mice after 7 days CFA. **(C)** Relative mRNA-expression levels for the Bdnf in lumbosacral SC of untreated control mice (control) and in ipsi- (ipsi) and contralateral (contra) lumbosacral SC of C57BL/6 and Sphk2^−/−^ mice after 7 days CFA. **(D)** Relative mRNA-expression levels for the Nos2 (iNos) in lumbosacral SC of untreated control mice (control) and in ipsi- (ipsi) and contralateral (contra) lumbosacral SC of C57BL/6 and Sphk2^−/−^ mice after 7 days CFA. **(E,F)** Relative mRNA-expression levels for the Il-1β and Il-6 in lumbosacral SC of untreated control mice (control) and in ipsi- (ipsi) and contralateral (contra) lumbosacral SC of C57BL/6 and Sphk2^−/−^ mice after 7 days CFA. Values represent mean ± SEM, *n* = 5–8, one-way ANOVA, Bonferroni *post hoc* test, *p < 0.05, **p < 0.01.

To determine whether nocifensive behavior differences could be related to changes in the signaling response to inflammation, we analyzed the expression of molecular markers involved in the spinal response to peripheral inflammation. The responses to inflammation in the periphery changed the expression of marker molecules at the level of the SC but not the relative expression levels for Sphk1. The Sphk1 mRNA-expression in the lumbosacral SC did not change in Sphk2-deficient mice 7 days after CFA injection (Figure 8) whereas injection of CFA into the left hindpaw induced a significant increase in mRNA expression for P_2_X_4_ receptors, Bdnf and Nos2 (iNOS) in the ipsilateral SC of C57BL/6-wt mice compared to the ipsilateral SC of Sphk2-deficient mice Those changes in relative mRNA expression levels were absent in the SC of Sphk2-deficient mice (*n* = 6–9, one-way ANOVA, Bonferroni’s *post hoc* test, Figure 8). Relative expression levels of the interleukins IL-1β and IL-6 were not significantly different between strains (Figure 8).

### Sphk2-Deficiency Increases Iba-1 Positive Area in Response to Chronic Inflammation in the ipsi- and Contralateral Lumbosacral Dorsal Horn

**Figure 9:**
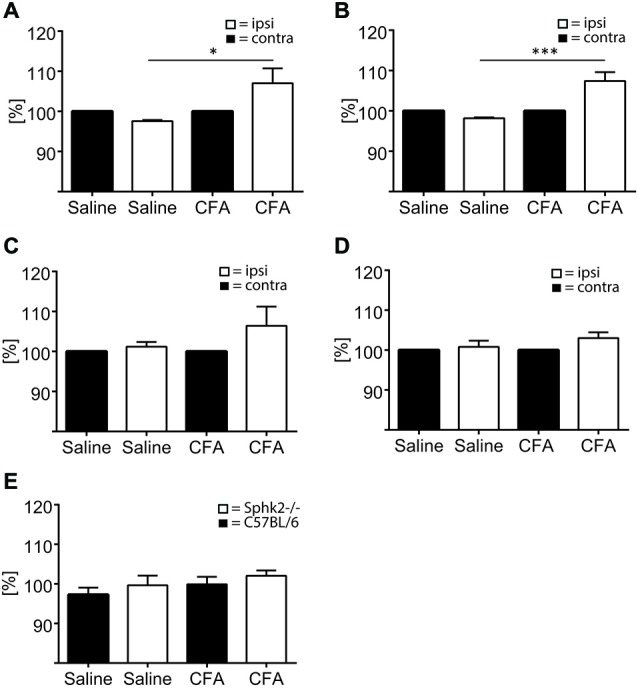
**Microglia and astrocytes in the dorsal horn in C57BL/6 and Sphk2^−/−^ mice in response to chronic inflammation**. Areas of the ipsi- and contralateral (contra) SC dorsal horn with IR for GFAP **(A,C)**, Iba-1 **(B,D)** and positive staining for Dapi **(E)** in C57BL/6 **(A,B,E)** and Sphk2^−/−^ **(C, D)** lumbosacral SC 7 days post injection of CFA (CFA) or saline (saline) into the left hindpaw. Values represent mean ± SEM, *n* = 5, one-way ANOVA, Bonferroni’s *post hoc* test, *p < 0.05, ***p < 0.001.

Positive labeling for astrocytes (GFAP) and microglia (Iba-1) could be observed throughout the SC dorsal horn. The SC of untreated control animals and animals injected with saline showed no difference of Iba-1 staining between ipsi- and contralateral sides and between C57BL/6-wt and Sphk2^−/−^ mice (Figure 9). Sections of C57BL/6-wt mice showed 7 days post CFA injection significant increases in areas with IR for GFAP and Iba-1 in ipsilateral lumbosacral SC dorsal horn (Figure 9, one-way ANOVA, Bonferroni’s *post hoc* test, 60–80 sections, *n* = 5). This increase was absent in Sphk2^−/−^ mice (Figure 9). The increase was reflected by overall increased IR ipsilateral compared to contralateral over the entire length of lumbosacral SC. However, immunoreactivities varied in between different segments along the lumbosacral SC with differences of up to 60% between sections in all animals. The total number of nuclei in the dorsal horn was not different between ipsi- and contralateral sides, strains and treatment.

## Discussion

Our study has shown that activation of the Sphk2 isoform is part of nociceptive signaling in response to acute and CPI at the level of the SC. We demonstrated for the first time that Sphk2-deficient mice respond earlier but with decreased intensity to acute inflammation and develop bilateral mechanical hypersensitivity in response to chronic inflammation. The behavioral changes to CPI were accompanied by the novel finding of a bilateral reduction in spinal S1P levels. Sphk2-deficient mice also failed to respond to CPI at the level of activation of glia and increased expression of glia-related mRNAs.

Studies in gene-deficient mice have been shown to generate fundamental insight in the signaling pathways underlying chronic and neuropathic pain. Unfortunately knock-out of a gene can cause compensatory upregulation of other related genes. Deletion of Sphk2^−/−^ has been shown to induce Sphk1 in the circulation (Kharel et al., 2012; Liang et al., 2013). To determine the impact of Sphk1 on the S1P content in the mouse SC and to rule out a compensatory regulation of Sphk1 in Sphk2^−/−^ mice we measured S1P levels, Sphk activities and relative mRNA expression levels for the Sphk isoforms. Although Sphk1 activity was not different between Sphk2^−/−^ and C57BL/6-wt SC, the absence of Sphk2 but not Sphk1 strikingly reduced S1P levels. This suggests an absence of a compensatory mechanism in the regulation of Sphk1 and supports the notion that the Sphk2 isoform is the predominant source of S1P in the SC. Our results are corroborated by recent studies using a different Sphk2-deficient mouse strain where a similar reduction in spinal S1P levels was shown (Zhang et al., 2015).

The Sphk2 isoform being the predominant Sphk in the SC is significant, as recent evidence indicates it may play a critical role in nociceptive signaling. Mice with genetic ablation of Sphk2 but not Sphk1 showed signs of thermal hyperalgesia in response to Zymosan injection (Coste et al., 2008a). Our study suggests the involvement of Sphk2 activation in the processing of acute inflammation but not acute pain. Whereas the acute response to injection of formalin and subsequent activation of sensory fibers in the skin (1^st^ phase) was identical in C57BL/6 and Sphk2^−/−^ mice, this was not the case for the 2^nd^ phase. Absence of Sphk2 caused a significantly earlier response peak (Figure 3). The 2^nd^ phase of the formalin test is thought to be related to central sensitization (Hunskaar and Hole, 1987; Yaksh et al., 2001) at the level of the SC indicating a role of Sphk2-generated S1P at the level of SC and/or higher brain centers although a recent publication questions it and relates both phases to the activity of DRG neurons (Fischer et al., 2014). These differences in response to acute inflammation between Sphk2^−/−^ and C57BL/6 mice are not based on a difference in the basal sensitivity to mechanical stimuli since the response to lower formalin concentration and the initial response to 2% formalin were similar between strains.

These behavioral changes were not accompanied by differences in IR for glial markers or NeuN. Our immunohistochemical analysis of the overall number of cells, of neurons and of GFAP-positive astrocytes and Iba-1 positive microglia in the dorsal horn also suggests that deficiency in Sphk2 does not lead to loss of neurons or glia. The absence of changes in microglia reflected by Iba-1 IR in response to 1 h acute inflammation is in line with experiments that showed activation of microglia in response to formalin was not present within the first hour and started at later time points post injection (Lin et al., 2007).

Since mechanical hypersensitivity in response to chronic inflammation is likely to be mediated via changes in pain processing at the level of the SC (see review Latremoliere and Woolf, 2009) we used the CFA model of CPI to further investigate the role of Sphk2 at the SC level.

Our observation of bilateral mechanical hypersensitivity i.e., enhanced hypersensitivity not only in the ipsilateral but also contralateral hindpaw of Sphk2-deficient mice suggests that Sphk2 is involved in the communication between ipsi- and contralateral side of the SC dorsal horn. We found no differences in oedema size over time and no difference in the extent of inflammation or ipsilateral PWL between C57BL/6 and Sphk2^−/−^ mice which indicates no obvious difference at the level of the inflammation in the skin and the response to peripheral chronic inflammation at the level of primary nociceptive neurons. Similarly Linke et al. (2012) found no differences in the response to Zymosan in Sphk2-deficient mice in the first 48 h but did not report on contralateral effects. Contralateral pain is still not well understood. Acute pain is usually well mapped to the site of peripheral injury or inflammation. However, in wide-spread chronic pain, pain and mechanical allodynia (pain perception upon non-noxious mechanical stimulation) is experienced on unaffected sites of the body even contralateral to the injured or inflamed side. Contralateral allodynia usually has a delayed onset and is usually weaker compared to pain on the ipsilateral side (Milligan et al., 2003; Chang and Waxman, 2010) and is regularly observed in different animal models of neuropathic and inflammatory pain including burn injury (Leclercq et al., 2011), ligation and inflammation of the sciatic nerve, or peripheral inflammation after injection of carrageenan or CFA (Milligan et al., 2003; Schreiber et al., 2008; Gao et al., 2010). Since peripheral nociceptors on the contralateral side are neither stimulated nor damaged, it is unlikely that they be the primary source of contralateral allodynia. In our experiments the initial response to injecting formalin injection and the mechanical sensitivity before CFA injection were not different between C57BL/6 and Sphk2^−/−^ mice. This indicates that peripheral nociceptors are unlikely responsible for the contralateral hypersensitivity. Previous studies using Sphk-deficient mice showed that mice with deficiency for Sphk2 but not Sphk1 showed significant lower pain thresholds to heat (Coste et al., 2008b). However, the study did not investigate mechanical thresholds and did not comment on bilateral effects.

Interestingly Sphk2^−/−^ SC contained significantly reduced S1P levels which confirms a recently published study (Zhang et al., 2015). However we could show that under chronic inflammatory conditions S1P-levels drop in C57BL/6 SC to levels of Sphk2-deficient mice. A reduction of spinal S1P levels had been shown in response to formalin and carrageenan but only investigated the response up to the first 9 h (Coste et al., 2008a). Here we show for the first time bilateral S1P reduction under chronic inflammatory conditions and provide indirect evidence that suggest the inhibition of mainly Sphk2 as the reason for reduced S1P levels. Nevertheless an involvement of Sphk1 cannot be ruled out since S1P levels also dropped in Sphk2-deficient mice.

Recent results suggest that in addition to neurons, microglia might also be involved in the development of contralateral hypersensitivity (Choi et al., 2015). The study identified that inhibition of astrocyte activation via microglia prevents bilateral hypersensitivity in response to peripheral inflammation. The involvement of microglia in the process is intriguing. In our study we found that the increase in mRNAs for several markers that are characteristic for activated microglia in response to peripheral inflammation was absent in mice with deficiency in Sphk2.

One of those markers is the ATP-receptor subtype P_2_X_4_. The generation and presence of P_2_X_4_ receptors in microglia is important for the development of mechanical allodynia and they are upregulated in microglia in response to peripheral inflammation (Tsuda et al., 2003). This upregulation is important for subsequent Bdnf production and release from microglia as it has been shown in response to peripheral nerve injury (Ulmann et al., 2008). Synthesis and release of Bdnf from spinal microglia causes the disinhibition of GABAergic interneurons (Trang et al., 2012). The connection of P_2_X_4_ and Bdnf is supported in our study at the transcriptional level. Our studies showed absence of increases in P_2_X_4_ and Bdnf in Sphk2^−/−^ SC. This furthermore indicates a prominent role of Sphk2 activation and S1P in those signaling pathways and also suggests the involvement of microglia. We also found absence of an increase in relative mRNA expression for the NOS2 isoform, iNOS. Microglia express iNOS (Sung et al., 2012) and iNOS is increased in response to inflammation but there seems to be no direct impact of iNOS on the response to CFA since complete knock-out of iNOS had no effect on mechanical hypersensitivity in response to peripheral inflammation (Boettger et al., 2007). The increase in iNOS mRNA might be related to signaling from higher brain centers. Reduction of μ-opiod receptor expressing cells in the medulla reduced the expression of iNOS in the dorsal horn of rats in response to CFA injection (Carr et al., 2014). In our study, absence of the peripheral inflammation-dependent increase in mRNA expression for molecules that are predominantly expressed in microglia was accompanied by absence of increased IR for the marker of microglia Iba-1. Based on the findings one hypothesis is, that S1P, generated from Sphk2 in SC microglia is part of the signaling that leads to activation of microglia and Sphk2 and microglia are involved in the communication between ipsi- and contralateral SC. The process of S1P-mediated activation of microglia with subsequent synthesis of pro-inflammatory mediators has been shown in experiments conducted to demonstrate the connection between chronic morphine administration and S1P signaling in rats (Muscoli et al., 2010).

Thus, our data strongly support the notion that the Sphk2 isoform is a key molecule involved in pain processing at the level of the SC dorsal horn. Furthermore, our data suggest a direct or indirect involvement of Sphk2 function in the glial response to peripheral inflammation, as well as in the inhibition of contralateral allodynia.

## Conflict of Interest Statement

The authors declare that the research was conducted in the absence of any commercial or financial relationships that could be construed as a potential conflict of interest.
